# A Retrospective Evaluation of 5 Years of Clinical Results of Metal–Ceramic vs. Monolithic Zirconia Superstructures in Maxillary All-on-4^TM^ Concept

**DOI:** 10.3390/jcm13020557

**Published:** 2024-01-18

**Authors:** Mustafa Ayna, Søren Jepsen

**Affiliations:** Department of Periodontology, University Hospital Bonn, University Bonn, 53127 Bonn, Germany; sjepsen@ukb.uni-bonn.de

**Keywords:** immediate loading, metal–ceramic, zirconia

## Abstract

The aim of the current study was to present the clinical and radiological outcomes of monolithic zirconia superstructures compared to the metal–ceramic ones in the All-on-4 concept for the prosthetic rehabilitation of the maxillae. A total of 30 patients were subdivided into groups according to their superstructure type (metal–ceramic (n = 15) or monolithic zirconia (n = 15)). All implants were functionally loaded within 24 h after insertion with provisional acrylic superstructures. Prosthetic complications, marginal bone loss, plaque accumulation, probing pocket depth, bleeding on probing, and bite force were documented over a period of 5 years. Marginal bone loss around the implants of the ceramic group remained well over the five years (1.21 ± 0.23 mm). However, marginal bone loss was significantly lower around the implants in the monolithic zirconia group (0.22 ± 0.14 mm) (*p* < 0.001). Bleeding on probing, plaque accumulation, and probing pocket depth values were correlated with marginal bone loss. Among all evaluated parameters, no differences could be detected in terms of the angulation of the implants. Detachment or chipping was observed in seven cases in the metal–ceramic superstructure group. In all cases, dentures were removed and repaired in the laboratory. In the monolithic zirconia group, chipping was detected after one year in two cases, after two years in four cases, and after five years in one case and could be managed by polishing in situ. Monolithic zirconia superstructures presented superior results regarding the parameters evaluated.

## 1. Introduction

In recent years, CAD/CAM technology has allowed the use of rapid manufacturing processes to improve the efficiency of implant-supported treatment options [1]. As a result, the application of metal-free restorations as restorative materials for implant-supported superstructures has gradually increased [2]. However, despite the wide range of restorative materials available on the market and the variety of therapy options today, the selection of the most appropriate material to obtain the best clinical results and meet the patients’ expectations is still challenging, and it remains difficult for dental professionals to determine the best restorative solution for each therapy concept.

It has been suggested that the main advantages of zirconia in implant-supported dental rehabilitation are reduced treatment costs and treatment time [1,2,3]. Another advantage in comparison to the metal–ceramic superstructures is proclaimed to be improved aesthetic outcomes, which have been attributed to the superior peri-implant soft tissue profile [3].

Within the last two decades, the use of full fixed prostheses mounted immediately after insertion on two straight mesial and two tilted distal implants—which is called the All-on-4 concept—has become nearly a standard therapy option for the rehabilitation of the edentulous jaws, with predictable clinical outcomes [4,5]. The literature shows very high survival rates for this concept; however, the peri-implant health status, complication rates, and reparatory costs could vary with the superstructure type selected [6]. This concept includes basically two different types of prosthetic solutions regarding the final protocol: a metal–ceramic fixed prosthesis with ceramic veneers or a fixed acrylic resin prosthesis with a metal framework and acrylic resin prosthetic teeth [5,7,8]. Therefore, to date, the most investigated restorative material in the All-on-4 is the metal–ceramic vs. acrylic superstructures, and the long-term behavior of monolithic zirconia superstructures in the All-on-4 concept remains an open question.

The long-term clinical outcomes of monolithic zirconia have become the main subject of various studies. However, the long-term outcomes have yet to be elucidated in the All-on-4 concept. The aim of the current study was to present the clinical and radiological outcomes of monolithic zirconia superstructures compared to the metal–ceramic ones in the All-on-4 concept for the rehabilitation of the edentulous maxillae. The null hypothesis states that there are no differences regarding the clinical and radiological outcomes between monolithic zirconia and metal–ceramic superstructures.

## 2. Materials and Methods

### 2.1. Study Design

Data and clinical documentation of the patients who were treated between August 2016 and July 2017 for the rehabilitation of their edentulous upper jaw with immediately functionally loaded implant-supported fixed prosthesis according to the All-on-4 concept were collected retrospectively. Patients were divided into subgroups according to the superstructure type (monolithic zirconia or metal-supported ceramics). Inclusion criteria were:

- Natural dentition or tooth/implant supported fixed prosthetics of the mandible;
- Regular attendance of dental recall appointments with intervals of 6 months (according to local regulations [9], irregular attendance during the COVID-19 time was ignored).

Patients with the following conditions were excluded [6,7]:

- Uncontrolled general diseases and/or having possible local and/or systemic contraindications for implant surgery;
- Neurological diseases impairing maintenance of oral hygiene, medications negatively affecting the implant healing processes (antiresorptive, corticosteroids, serotonin reuptake inhibitors, etc.);
- The history of active inflammatory processes (osteomyelitis) or neighboring neoplasms at the maxilla or maxillary sinuses;
- Radiotherapy to the head and neck region;
- Temporomandibular-joint-related symptoms;
- Bruxismus;
- Bone and/or soft tissue augmentations;
- Nicotine/drug abuse;
- Previously operated maxillary sinuses.

### 2.2. Preoperative Measurements

Prior to the placement of the implants, vertical dimensions were determined, occlusal registrations were conducted, and a denture for the upper jaw was finished by a dental laboratory, which was modified and immediately mounted to serve as a provisional fixed restoration postoperatively. Preoperative photographs were made to record the aesthetic aspects such as the smile line, lip raised, and in resting position.

### 2.3. Surgery

**Figure 1:**
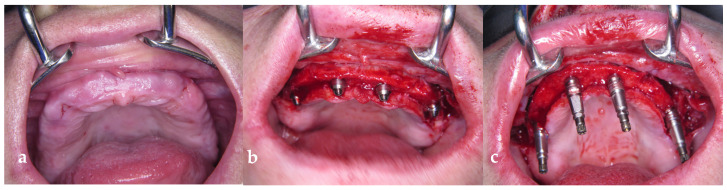
(**a**) Initial clinical situation. (**b**) Insertion of the implants. (**c**) Impression copings mounted on implants.

Surgical interventions were performed by M.A. under local anesthesia and in some cases under conscious sedation with I.V. benzodiazepine (0.17 mg/kg). All patients received four implants (HiTec LGI, Herzliya, Israel) according to the All-on-4 treatment concept: two straightly placed mesial (placed in region 12 and 22) and two angulated distal implants (Figure 1a–c, placed in regions 15 and 25). Implant sizes were 13 mm for the straight and 16 mm for the angulated implants, respectively, all with a diameter of 4 mm. Briefly, after raising a mucoperiosteal flap with releasing incisions on the vestibular aspect in the distal to the molar and the midline area, the whole maxillary alveolar crest was exposed. In the presence of a high smile line, bone level was reduced with an ostectomy. After the identification of the anterior bony wall of the maxillary sinus through a bony window of 3 mm, the placement was started with the posterior implants with the aid of a special guide (Edentulous guide, Nobel BiocareTM, Göteborg, Sweden). The insertion of all implants followed standard procedures according to the manufacturer guidelines; however, a peak insertion torque of at least 35 N/cm should be reached to allow an immediate functional loading concept. After placement of the implants, impression copings were placed, and a primary closure was conducted via 3–0 nonresorbable sutures. The functional loading was performed within 24 h after insertion. Postoperative instructions, such as the avoidance of vigorous mouth rinsing, immediate use of ice packs in the first 24 h, upright sitting, a liquid or soft diet for the first 24 h, avoidance of eating crunchy foods, and restriction of physical activities on the day of surgery, were given. Ice packs were placed on the outside of the face where the implants were placed. Ice was used for the first 48 h to decrease swelling by applying it as continuously as possible. Antibiotics (amoxicillin 875 mg/clavulanic acid 125 mg) were given 1 h prior to surgery and two times a day for 5 days thereafter. For the patients with penicillin allergy, clindamycin 600 mg was prescribed with the same posology. The sutures were removed 7 days after surgery.

### 2.4. Provisional Prosthetic Procedure

**Figure 2:**
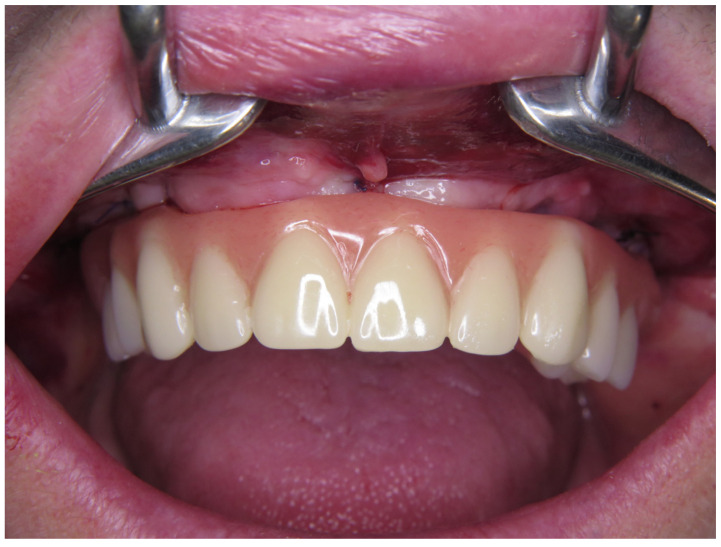
Provisional acrylic prosthesis in situ.

Impressions were taken with an open-tray technique. After placement of the impression copings, the positions were determined with a surgical pen, and drill holes were created on the prefabricated impression tray. Implants replicating multiunit abutments were connected to the impression copings, screws were loosened, and the impression tray was removed with impression copings. The impression copings were removed from the material, and temporary abutments were placed on the implant replicas in the preliminary model ex situ. The implants were covered with temporary healing caps. Within 24 h, a screw-retained acrylic prosthesis was manufactured at the dental laboratory and mounted on the implants (Figure 2). To ensure an ideal occlusal relationship, both centric and lateral contacts and discursions were controlled, and the prosthesis was adjusted in situ.

### 2.5. Final Prosthetic Procedure

**Figure 3:**
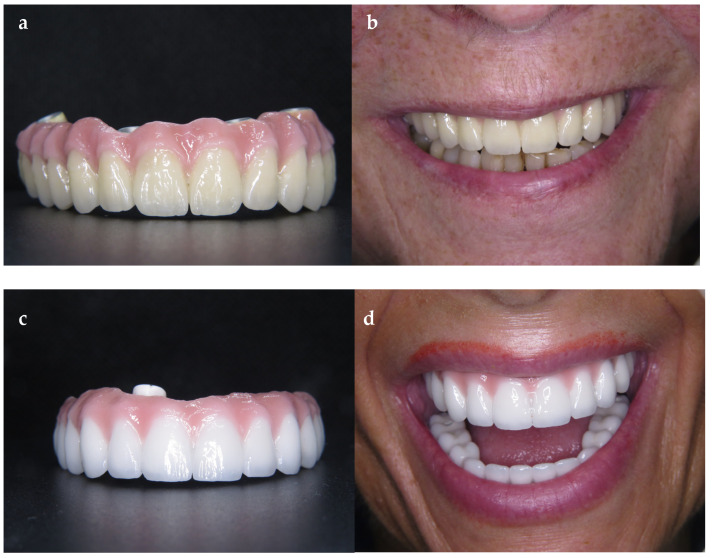
(**a**,**b**) Metal–ceramic implant-supported fixed prosthesis; (**c**,**d**) monolithic zirconia.

The final prosthetic rehabilitation was performed 3 months after surgery. The patients were assigned to subgroups according to their own preference of the superstructure. For the patients who were treated with metal–ceramic superstructures, a chrome–molybdenum framework was produced with CAD/CAM (Figure 3a,b). The monolithic zirconia prostheses (Figure 3c,d) with a 1-piece design were all fabricated with the same brand of multilayered zirconia (Kuraray Europa GmbH, Hattersheim, Germany) by using the protocols recommended by the manufacturer. In both groups, the length of the posterior cantilever was determined according to 1.5–2 xA-P-spread rule [5], which allows a 10–12 mm distal cantilever extended to the molar area. The tissue surface of all superstructures was rounded, smoothened, and polished to avoid food impaction and to favor the patient’s mouth hygiene.

### 2.6. Outcome Parameters

Implant survival, prosthetic complications, marginal bone loss, probing pocket depth in mm (PPD), bleeding on probing (BOP), and plaque accumulation were documented for each implant during a period of 5 years. To ensure the accuracy and reproducibility of each parameter except the radiological assessment, the superstructures were removed. The bite force and its distribution were measured preoperatively, immediately after functional loading, and during the whole examination period.

**Figure 4:**
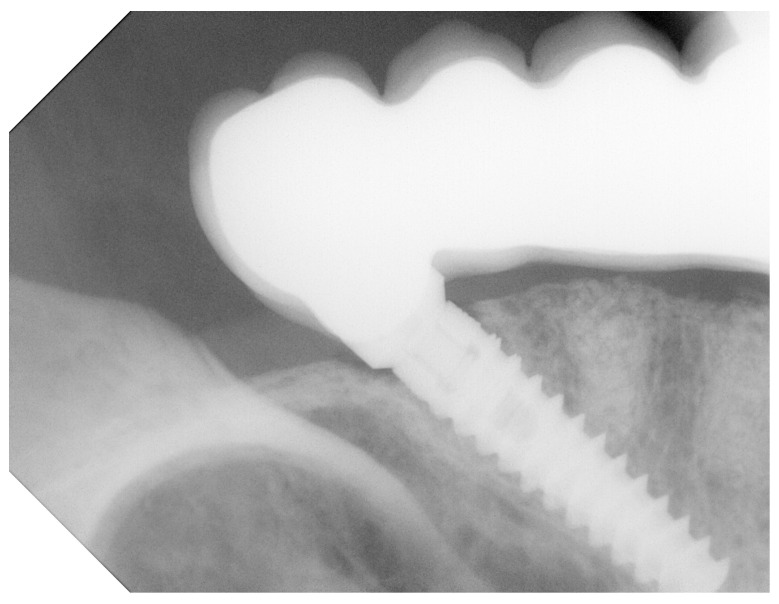
Marginal bone loss was evaluated by measuring the limbus alveolaris around the implants, by using standard right-angle parallel technique with single digital radiographs.

Marginal bone loss was evaluated by measuring the limbus alveolaris around the implants by using the standard right-angle parallel technique with single digital radiographs (Figure 4), as described by Brägger [10,11]. The radiographs were scanned at 600 dpi (Trophy RVG UI USB Sensor, KODAK 5.0 software, Carestream, Stuttgart, Germany). The bone level was comparatively assessed with image analysis software (IC Measure, Version 2, The Imaging Source Europe GmbH, Bremen, Germany).

PPD was measured with a calibrated periodontal probe (Hu-Friedy, Chicago, IL, USA). BOP was documented at four sides (mesial, buccal, distal, and palatinal) according to the modified Sulcus Bleeding Index, described by Mombelli et al. [12] as follows:
*The deepest pocket bleeding probing*“1”*The deepest pocket none bleeding by probing*“0”

Plaque accumulation was evaluated using the Plaque Index according to Mombelli et al. [12] with the following quantification:
*No detection of supragingival plaque*“0”*Plaque only recognized by running a probe across the smooth*“1”*Plaque can be seen by the naked eye*“2”*Abundance of soft matter*“3”

**Figure 5:**
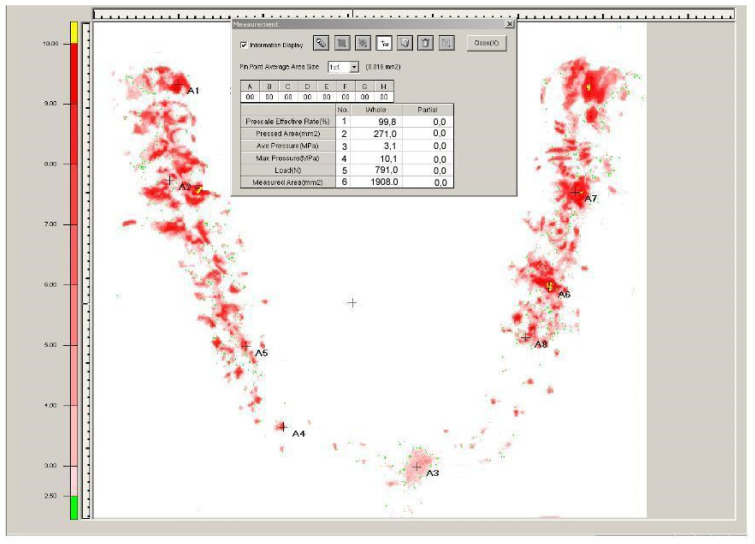
Analysis of the bite force with Dental Pre-scale 50H type R and Occluzer FPD-703 (Fuji Photo Film Co., Tokyo, Japan). The distribution shows a well-balanced occlusal load on the molar area, which corresponds to the natural dentition.

Bite force and its distribution were assessed via a pressure-sensitive film (Dental Prescale 50H-R-FPD-703; Fuji Photo Film Co., Tokyo, Japan) (Figure 5).

### 2.7. Statistical Analysis

ANOVA was used to establish the sample size needed to compare the contrast null hypothesis H_0_: μ1 = μ2 resulting in 80% power with a confidence level of 5%. Data were analyzed using “Python 3.6.15” (open-source programming language). At first, mean and standard deviation for each group were calculated. The Shapiro–Wilk test was performed to assess the distribution of the parameters. Before the statistical evaluation of the groups, Levene’s test was used to determine homogeneity of variances. The parametric and nonparametric methods were an independent T test and a Mann–Whitney U test for group differences and discrete parameters, respectively. The level of significance was set at *p* < 0.05. The Pearson correlation coefficient was calculated to analyze the relationship between scale variables.

## 3. Results

A total of 30 patients (16 women, 14 men) with a mean age of 64 ± 9.4 years were recruited. The size of both subgroups was equal (n = 15). In total, 120 implants were inserted. Due to the local regulations during the COVID-19 pandemic, the attendances between 2019 and 2020 could not be performed and the postoperative data of years 1, 2, and 5 were included.

### 3.1. Implant Loss

The implant survival loss was 100%. In addition, no complications such as infection of the sinus, abscess, or fistula were detected.

### 3.2. Prosthetic Complications

The survival rates for both immediate and final prostheses were 100%. No major complications, such as the fracture of the framework, occurred. Dislodgement of the acrylic teeth of the provisional superstructure was detected in four cases, which was managed in situ. Discoloring of the acrylic teeth was observed in nearly all provisional acrylic superstructures.

Loosening of the multiunit abutment screw was seen in two cases at the implant in the metal–ceramic group and was resolved by retightening the abutment screw.

Detachment or chipping of the veneering material was observed in seven cases in the metal–ceramic superstructure group after one (n = 2), two (n = 3), and five years (n = 2) following the adjustment. In all cases, dentures were removed and repaired in the laboratory. In the monolithic zirconia group, chipping was detected after one year in two cases, after two years in four cases, and after five years in one case and could be managed by polishing in situ. No further prosthetic complications were documented during the follow-up period of five years.

### 3.3. Bone Loss

**Figure 6:**
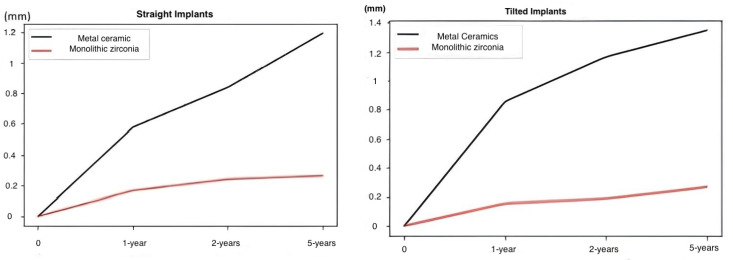
Bone loss was significantly lower around the implants in the monolithic zirconia group and the difference between two groups was statistically significant (*p* < 0.001). There were no significant differences in marginal bone loss between the straight and tilted implants.

In both subgroups, a progression of marginal bone loss was observed during the first year. However, after 1 year, the bone loss around implants of the metal–ceramic group was significantly higher (*p* < 0.001), both for tilted (1.33 ± 0.35 mm) and straight implants (1.15 ± 0.30) compared to the monolithic zirconia group (0.21 ± 0.11 mm for straight implants and 0.23 ± 0.15 mm for tilted implants, respectively). There were no significant differences in marginal bone loss between the straight and tilted implants (Figure 6).

### 3.4. Plaque Accumulation

**Figure 7:**
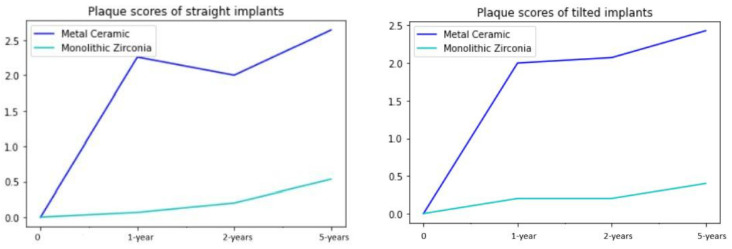
Plaque accumulation was significantly higher around the implants in the metal–ceramic group and the difference between two groups was statistically significant (*p* < 0.001). There were no significant differences in terms of plaque accumulation between the straight and tilted implants.

The Plaque Index showed that plaque accumulation was significantly lower around the implants in the monolithic zirconia group. The scores remained nearly unchanged following the one-year examination in both groups. There were no significant differences in terms of plaque accumulation between the straight and tilted implants (Figure 7).

### 3.5. Bleeding on Probing

**Figure 8:**
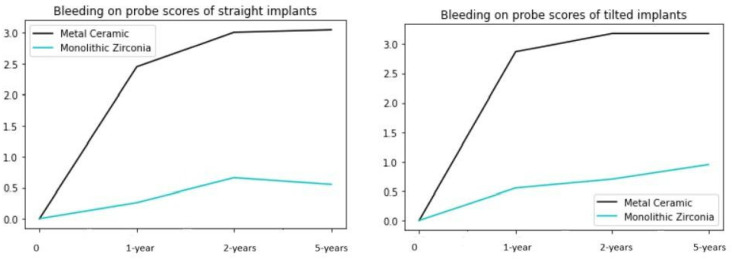
Bleeding on probing measurements around the implants revealed no statistically significant differences regarding the implant angulation. However, significantly higher values in the group with metal–acrylic superstructures were observed throughout the examination period over the monolithic zirconia group.

Bleeding on probing measurements around the tilted and straight implants revealed no statistically significant differences. However, significantly lower scores were observed around the implants in the monolithic zirconia group (Figure 8). The difference between the two subgroups was statistically significant (*p* < 0.001).

### 3.6. Probing Pocket Depth

**Figure 9:**
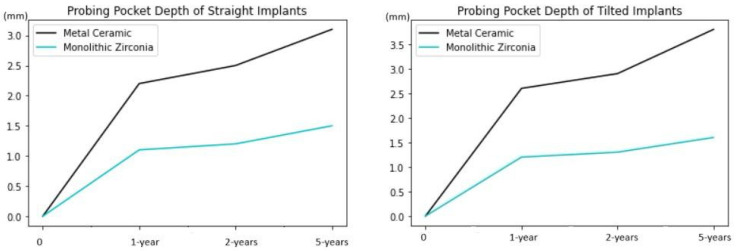
Comparative analysis of the probing pocket depths (mm) between metal–ceramic and monolithic zirconia groups regarding the implant regions revealed significant lower values in the monolithic zirconia group (*p* < 0.001).

PPD increased consistently and significantly over time in the metal–ceramic group. Significantly shallower pockets were found at the implants supporting the monolithic zirconia superstructures (Figure 9) There were no statistical differences between straight and tilted implants for both groups.

### 3.7. Bite Force

**Figure 10:**
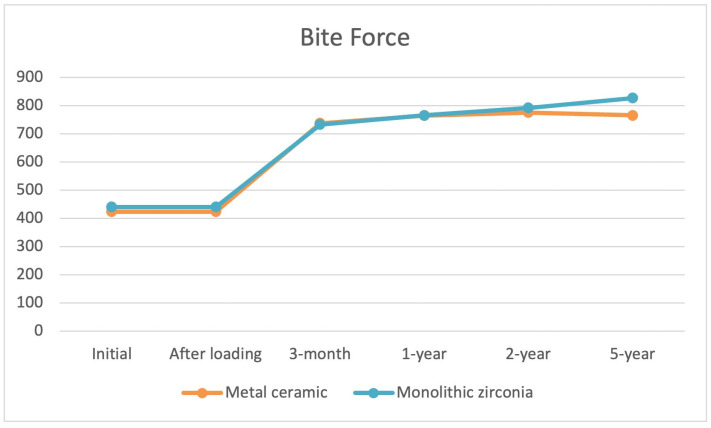
Occlusal force improved after immediate functional loading in both groups. An increasing difference in favor of monolithic zirconia began to evolve from 4 years onward; however, the difference was statistically not significant. The mean values of deviation regarding the differences in occlusal forces during the whole examination period was 651.41 N for the monolithic zirconia and 623.59 N for the metal–ceramic superstructures, respectively.

Bite force improved immediately after functional loading in both groups. An increasing difference in favor of monolithic zirconia superstructures began to evolve from 2 years onward; however, the difference was statistically insignificant (Figure 10).

## 4. Discussion

The results of this study reject the null hypothesis (H0) that there are no differences regarding the clinical and radiological outcomes between monolithic zirconia and metal–ceramic superstructures.

To the best of our knowledge, the clinical results of monolithic zirconia superstructures in the All-on-4 concept have not been evaluated until now. Barootchi et al. [13] compared the clinical outcomes of metal–acrylic and zirconia-implant-supported fixed prostheses and stated that zirconia fixed implant prostheses presented higher initial costs than metal–acrylic hybrids; however, they showed superior satisfactory outcomes, reduction in overall complications, and superior survival rates. This study has clearly shown that the advanced criteria for “success” in dental implantology [14], including bone resorption, were fulfilled throughout the sample after 5 years of observation for both groups. The results regarding the bone loss and inflammatory parameters in both groups were similar and parallel to those reported on the maxillary All-on-4 concept in the literature [15,16,17]. However, a slight superiority of the monolithic zirconia structures regarding the PPD and marginal bone loss could be observed.

Being highly wear-resistant, hard, and durable, it has been found that zirconia restorations do not follow the natural abrasions of and changes in the masticatory system [18]. However, Koenig et al. reported that there could be a weak link regarding the restoration support or the antagonist tooth, one hypothesis being that zirconia stiffness and lack of resilience do not promote occlusal stress damping [19]. Considering the bite force, this study has shown that the All-on-4 concept allows a sustainable improvement in functionality immediately after the integration of the superstructure in both groups. It is obvious that bruxism could result in both biological and mechanical failures in dental implantology and could have a direct effect on the therapy outcomes. Dreyer et al. described bruxism as a contributory factor in peri-implant infections [20]. Regarding the study design of the present study, the exclusion of the risk factor “bruxismus” might be viewed as a limitation.

The current study has clearly shown that the occlusal forces were significantly improved after functional loading. A retrospective assessment of the clinical performance of the complete oral rehabilitation of n bruxers treated with implants and teeth-supported restorations revealed that the survival rates of both veneered and nonveneered monolithic zirconia restorations and implants in patients with bruxism are excellent; however, veneered zirconia restorations tend to show chipping [21]. Similar results have been presented by Levartosky et al. [22], who concluded that the survival and success rates of monolithic zirconia restorations were nearly perfect; however, the veneered restorations showed a high rate of minor veneer chipping. This problem could be resolved via polishing in situ. Further studies are needed to determine whether zirconia superstructures should be favored in the All-on-4 concept for patients with bruxismus. However, regarding the minor mechanical complications of the superstructures, this study revealed equal results between metal–ceramic and zirconia subgroups. All mechanical complications with an esthetic aspect, such as chipping or detachment of the veneer, could be managed within one day at the dental laboratory for metal–ceramic superstructures. The most commonly seen complication in the zirconia group was chipping, which could be managed by polishing in situ without removing the superstructure.

A literature survey assessing the 5-year survival of metal–ceramic and zirconia superstructures showed that the survival rates of all types of all-ceramic prosthesis were lower than those reported for metal–ceramic fixed ones [23]. The incidence of framework fractures was significantly higher for ceramic groups, and the incidence for ceramic fractures and loss of retention secondary to the loosening of the screw was significantly higher for zirconia prostheses compared to metal–ceramic ones. Pelekanos et al. [24] described the combination of a monolithic zirconia with an anatomically shaped titanium framework to increase the flexural strength and fracture toughness and stated that this novel concept may be indicated to increase the clinical performance of full-arch prosthesis. Herklotz et al. [25] highlighted the accuracy of planning an immediate loading of full-arch zirconia restorations to avoid both mechanical and biological complications. In the current study, no major mechanical complications (implant fracture, fracture of the connection parts, or complete fracture of the framework) were detected. Loosening of the connection screw was detected in two implants in the metal–ceramic groups.

According to a survey [26] based on the data of 2039 complete arch fixed implant-supported zirconia prostheses, the survival rate of zirconia superstructures depends on:

- The quality of the material;
- Respecting the laboratory guidelines/protocols described by the manufacturer;
- Minding the emergence profile and the gap above the soft tissues for better access for maintaining oral hygiene;
- Avoiding excessive posterior cantilevers;
- The provision of an acrylic prosthesis to allow adjustment of function and esthetics prior to the definitive prosthetic treatment.

All the above-mentioned criteria particularly overlap with the principles of the All-on-4 concept: well-polished, a convex denture base, and a restricted cantilever length determined according to 1.5–2× antero–posterior spread rule, as previously described by Malo et al. [5], which allow a 10–12 mm posterior cantilever extended posteriorly and biologically highly compatible provisional prosthetics to create an ideal soft tissue profile.

Several studies [7,27] focusing on immediate loading revealed an increase in marginal bone loss from 5 years onward. Therefore, the current results might allow a mid-term consideration. Therefore, further studies with larger and more heterogenous sample sizes and long-term (>7 years) follow-up are needed to validate the findings of this study. Additionally, the highly selected study group regarding the exclusion of common risk factors in implantology such as bruxism, smoking, diabetes, etc., which could jeopardize peri-implant health, might be viewed as a limitation.

## 5. Conclusions

- The advanced criteria for “success” in dental implantology were fulfilled throughout the sample after 5 years of observation for both metal–ceramic and monolithic zirconia superstructures;
- Monolithic zirconia superstructures presented superior results regarding both clinical and radiological parameters evaluated herein;
- The mechanical complications in the monolithic zirconia superstructures were easily managed by polishing, whereas detachment of the veneer in the metal–ceramic group required an ex situ repair in the laboratory;
- No major mechanical complications could be observed during the follow-up period in either group.

## Data Availability

Data are available at Private Implantology Praxis Dr. Ayna, Duisburg.
